# Supplementary material for: Polyfunctionality and breadth of HIV-1 antibodies are associated with delayed disease progression
Source: PLoS Pathog. 2024 Dec 11;20(12):e1012739. doi: 10.1371/journal.ppat.1012739 (PMC11634010; doi:10.1371/journal.ppat.1012739)
Supplement: S1 Table — Abbreviations: AIDS: acquired immunodeficiency syndrome; n: number; SC = seroconversion; cp/mL = viral copies per milliliter. (DOCX) [file ppat.1012739.s001.docx]

**Supplementary Table 1**

| **Sample at 3 years post-seroconversion available** | | | | | |
| --- | --- | --- | --- | --- | --- |
| **Time between seroconversion and AIDS (years)** | | **<3** | **3-7** | **7-11** | **>11** |
| N in each group | | 28 | 147 | 66 | 71 |
| female % | | 12% | 6% | 8% | 11% |
| Age at SC (years) | median | 37 | 35 | 32 | 32 |
|  | range | 24-56 | 19-57 | 21-51 | 19-49 |
| Time since SC (months) | median | 36 | 36 | 36 | 36 |
|  | range | 28-38 | 26-52 | 21-56 | 30-54 |
| Setpoint viral load (cp/mL) | median | 56000 | 34000 | 12790 | 7400 |
|  | range | 1100-630000 | 250-1100000 | 215-410000 | 110-1300000 |
| Setpoint CD4+ T cell count (cells/µl) | median | 250 | 450 | 630 | 660 |
|  | range | 10-610 | 60-1390 | 111-1250 | 250-1820 |
| **Sample at 6 months post-seroconversion available** | | | | | |
| **Time between seroconversion and AIDS (years)** | | **<3** | **3-7** | **7-11** | **>11** |
| N in each group | | 20 | 59 | 21 | 15 |
| female % | | 18% | 5% | 17% | 36% |
| Age at SC (years) | median | 38 | 36 | 33 | 34 |
|  | range | 24-56 | 23-57 | 22-48 | 26-49 |
| Time since SC (months) | median | 5,8 | 5,4 | 6,0 | 5,9 |
|  | range | 4-8 | 4-9 | 4-11 | 4-11 |
| Setpoint viral load (cp/mL) | median | 44500 | 24000 | 9100 | 2815 |
|  | range | 1100-630000 | 1000-920000 | 215-240000 | 150-43000 |
| Setpoint CD4+ T cell count (cells/µl) | median | 250 | 450 | 600 | 760 |
|  | range | 10-610 | 210-1370 | 330-1250 | 250-1820 |
