# Supplementary figures and images for: Polyfunctionality and breadth of HIV-1 antibodies are associated with delayed disease progression
Source: PLoS Pathog. 2024 Dec 11;20(12):e1012739. doi: 10.1371/journal.ppat.1012739 (PMC11634010; doi:10.1371/journal.ppat.1012739)

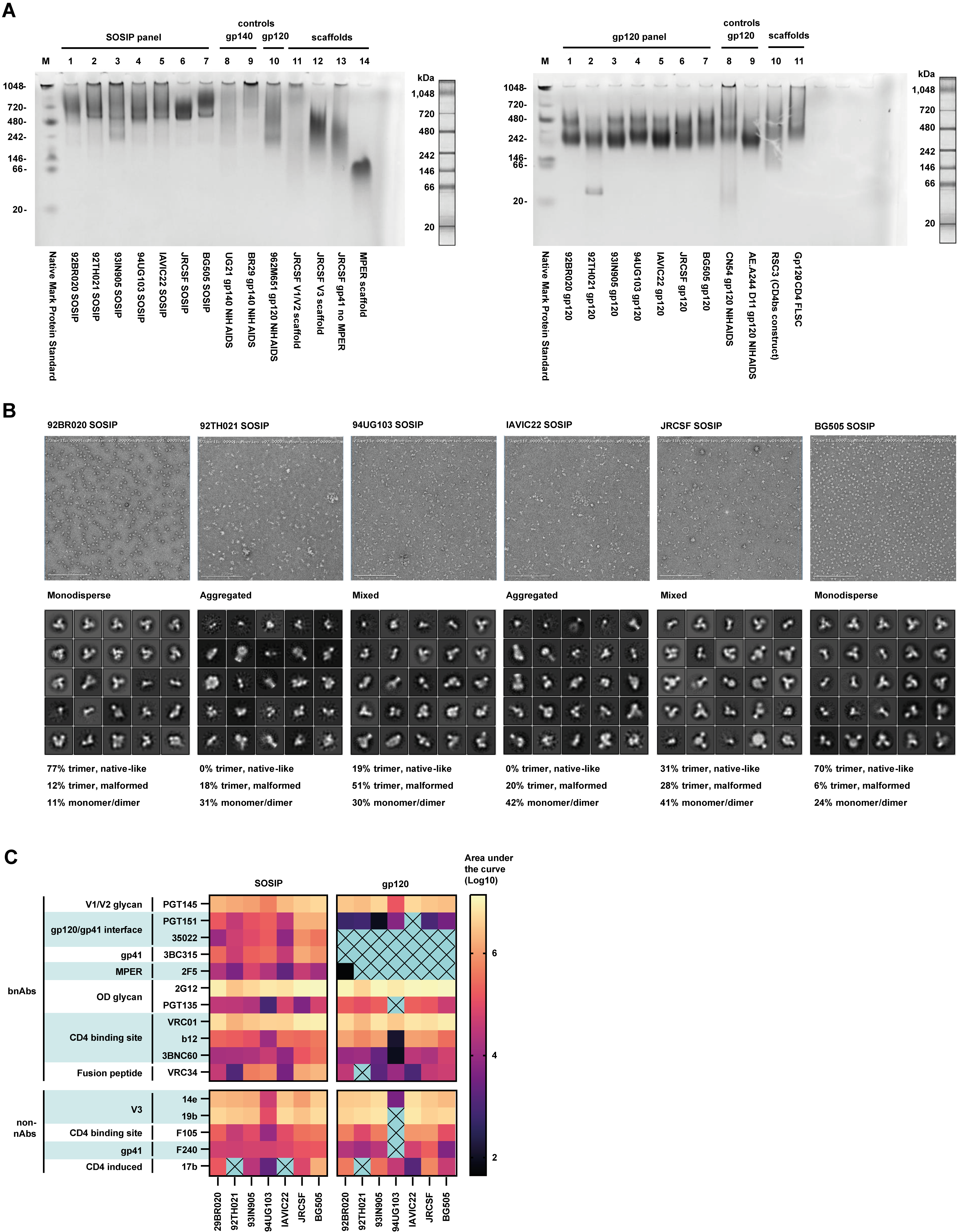

Supplement: S1 Fig — (A) Coomassie staining on BN PAGE gels loaded with protein antigens included in this study and control reagents. A marker (M) was included for a coarse estimation of size, but with preference of comparing the antigens with NIH-AIDS reagents; several monomer gp120 and gp140 control antigens were included. (B) Negative stain electron microscopy analysis of six SOSIP antigens. Micrographs (top) allow estimation of the degree of aggregation, while 2D classification (bottom) was used to estimate the amount of native-like trimer, malformed trimer and monomer/dimer. (C) Heatmap of Log10 transformed area under the curve of median fluorescence intensity values obtained by Luminex assay using the SOSIP and gp120 antigens indicated on the x-axis on beads and assessing binding of the monoclonal antibodies indicated on the y-axis. Epitope clusters recognized by the mAbs are indicated. The plot is subdivided in two categories: broadly neutralizing antibodies (bNAbs) and non-neutralizing antibodies (non-NAbs). All combinations were tested, a cross indicates that no binding was detected. Abbreviations: MPER: membrane-proximal external region. (TIF) [file ppat.1012739.s002.tif]

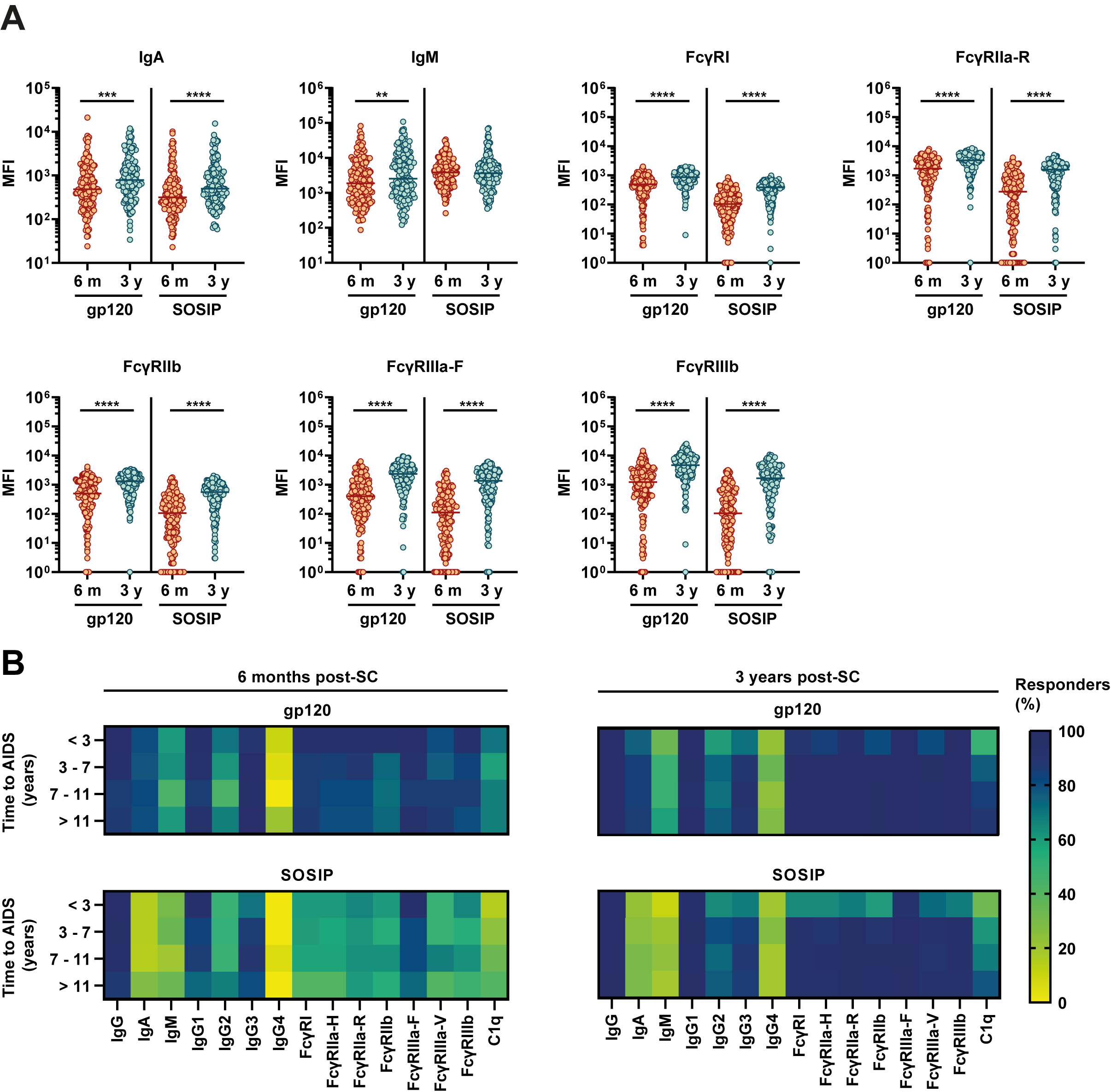

Supplement: S2 Fig — (A) 92BR020-specific antibodies in serum targeting SOSIP and gp120 antigens of IgA and IgM type and interacting with FcγRI, FcγRIIa-R (low affinity allelic variant R131), FcγRIIb, FcγRIIIa-F (low affinity allelic variant F158) and FcγRIIIb. Antibody responses are compared between the time point 6 months (6 m) after seroconversion (SC) and 3 years (3 y) after SC using a Wilcoxon matched-pairs signed rank test. Only individuals with samples at both time points (n = 166) are included in this subfigure. Data is expressed as median fluorescence intensity (MFI) measured by Luminex assay. (B) Responder rates in percentage are indicated by the heatmap colors. On the left, in serum of 166 participants at 6 months post-SC, compared between four groups based on time between SC and acquired immunodeficiency syndrome (AIDS) diagnosis (less than 3 years (n = 20), between 3 and 7 years (n = 59), between 7 and 11 years (n = 21) and more than 11 years (n = 15)). On the right, in serum of 382 participants at 3 years post-SC, split based on disease progression in four categories (less than 3 years (n = 28), between 3 and 7 years (n = 147), between 7 and 11 years (n = 66) and more than 11 years (n = 71)). Responses are compared for 15 different 92BR020 SOSIP- and gp120-specific antibody features. ** = P <0.01, *** = P<0.001, **** = P<0.0001. (TIF) [file ppat.1012739.s003.tif]

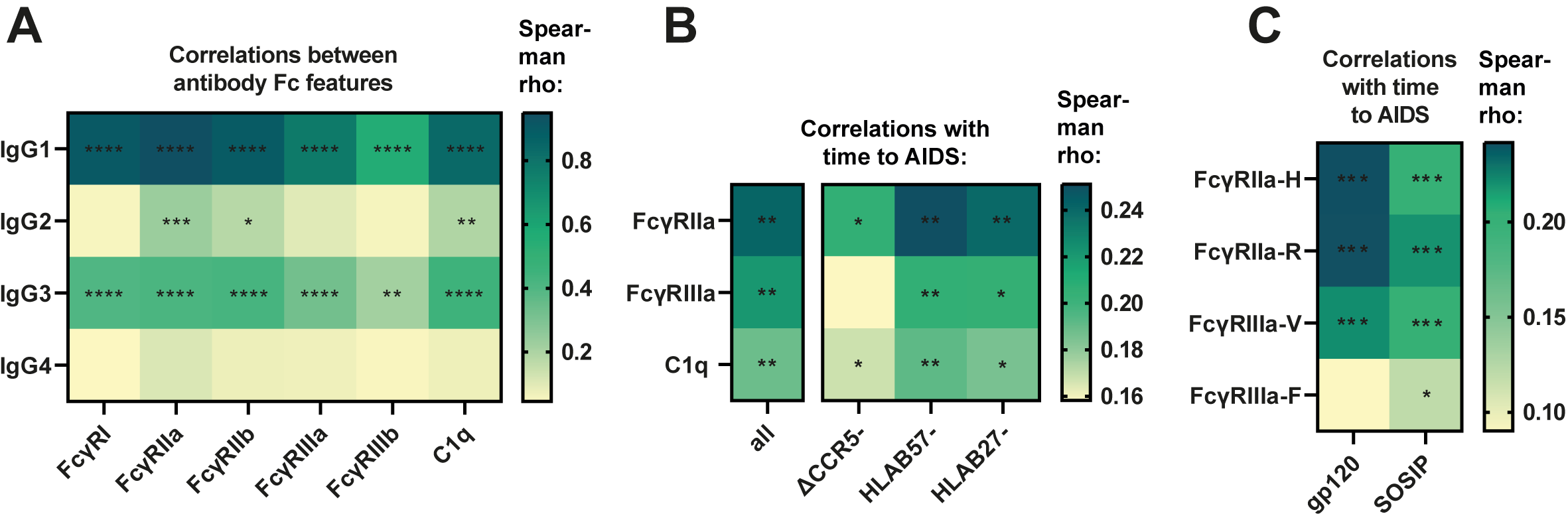

Supplement: S3 Fig — (A) Spearman correlations between the four subtypes of 92BR020 gp120-specific IgG and interaction of 92BR020 gp120-specific antibodies with all FcγRs and C1q for the full cohort (n = 382) at 3 years post-seroconversion (SC). (B) Spearman correlations between time between SC and acquired immunodeficiency syndrome (AIDS) diagnosis and 92BR020 gp120-specific antibodies interacting with FcγRIIa, FcγRIIIa and C1q. Results are shown for the full cohort with known time to AIDS (n = 312), individuals confirmed to not have the protective CCR5 deletion and with known time to AIDS (n = 231), individuals confirmed to not have the HLA-B57 gene and with known time to AIDS (n = 271) and individuals confirmed to not have the HLA-B27 gene and with known time to AIDS (n = 214). (C) Spearman correlations of time between SC and AIDS with 92BR020 gp120- and SOSIP-specific antibodies interacting with the two allelic variants of FcγRIIa and FcγRIIIa for the full cohort with known time to AIDS (n = 312). FcγRIIa-H and FcγRIIIa-V are the high affinity variants (FcγRIIa-H131 and FcγRIIIa-V158) while FcγRIIa-R and FcγRIIIa-F are the low affinity variants (FcγRIIa-R131 and FcγRIIIa-F158). (D) The color indicates the rho Spearman correlation coefficient and significant results are indicated by asterisks. The results were corrected for multiple comparisons using the Bonferroni-Holm method. * = P<0.05, ** = P <0.01, *** = P<0.001, **** = P<0.0001. (TIF) [file ppat.1012739.s004.tif]

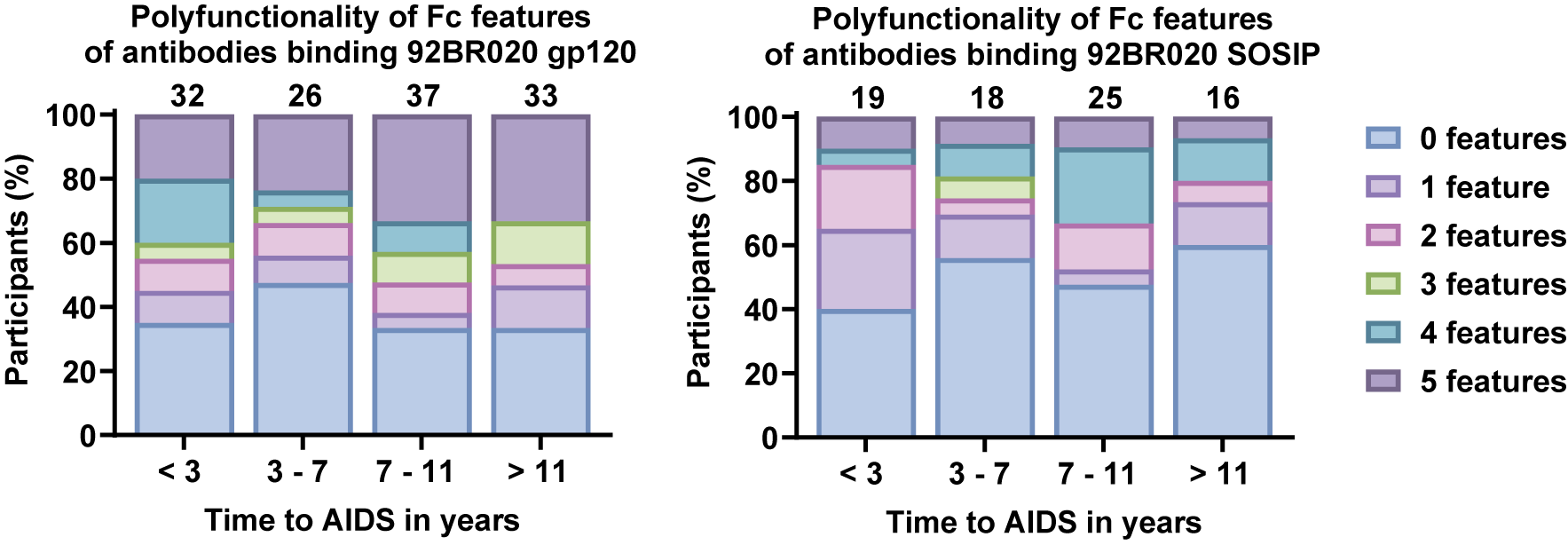

Supplement: S4 Fig — (A) Polyfunctionality was assessed by calculation of a polyfunctionality score and index at 6 months post-seroconversion (SC). We only assessed activating FcγR (FcγRI, FcγRIIa, FcγRIIIa, FcγRIIIb) and C1q interaction, and for each Fc feature we determined for each participant if there was a response that was higher than the median response of all responders in the cohort. The resulting scores (amount of features above the responder median, between 0 and 5) were plotted for the four groups based on time between SC and acquired immunodeficiency syndrome (AIDS) diagnosis (less than 3 years (n = 20), between 3 and 7 years (n = 59), between 7 and 11 years (n = 21) and more than 11 years (n = 15)) with one plot for gp120-specific responses and one plot for SOSIP-specific responses. In addition, a polyfunctionality index was calculated for each group as a quantitative measure of polyfunctionality. This is a value between 0 and 100 calculated by weighted addition of the percentage of individuals in each category based on the number of features, derived from Larsen et al.[54], see methods). This index is shown above each bar. (TIF) [file ppat.1012739.s005.tif]

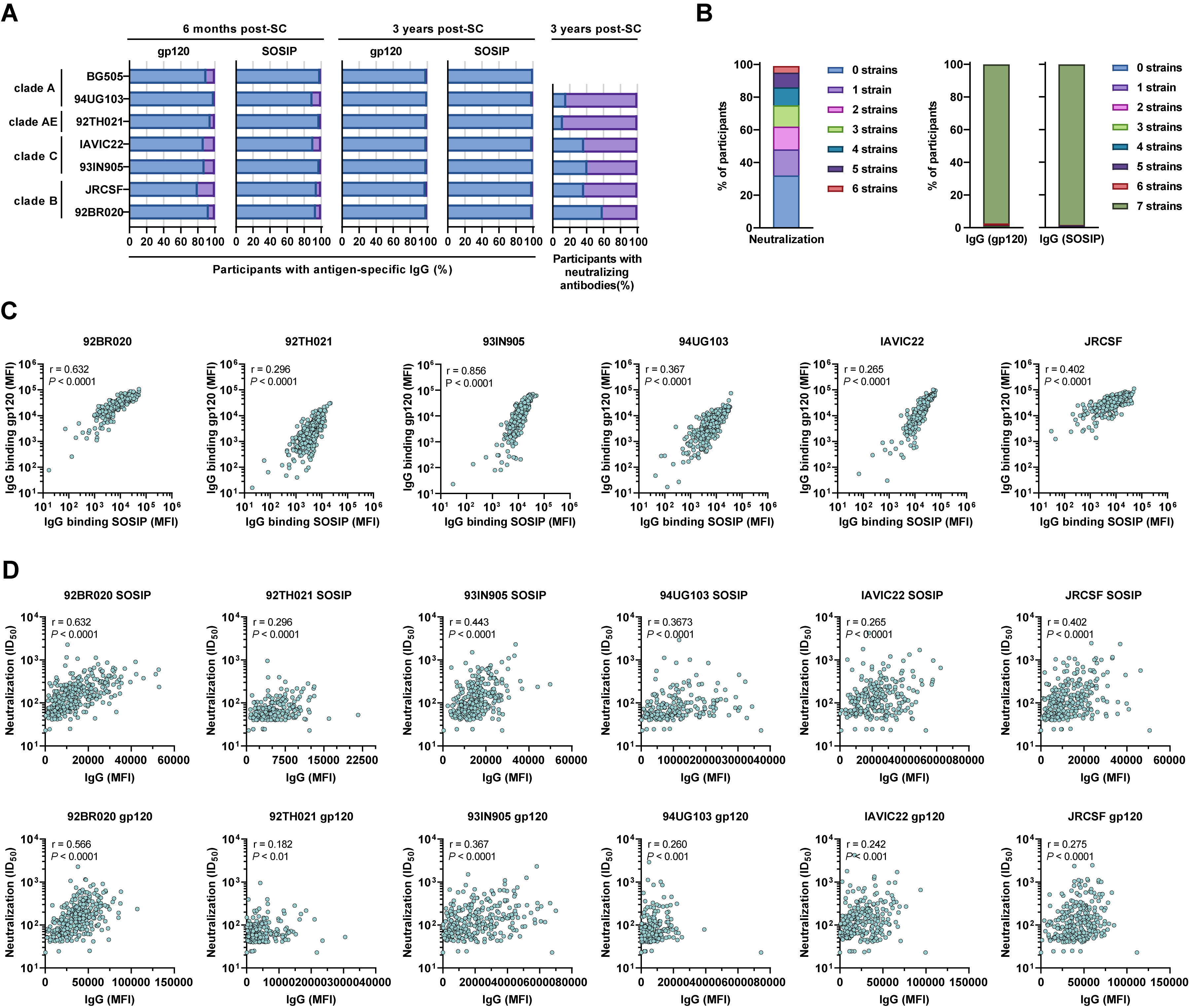

Supplement: S5 Fig — (A) On the left, responder rates in percent for IgG binding of seven HIV-1 strains. Shown for gp120-specific and SOSIP-specific responses, for 166 individuals at 6 months post-seroconversion (SC) and 382 individuals at 3 years post-SC. On the right, percentages of participants with detectable neutralizing antibodies in 382 individuals at 3 years post-SC. Neutralization assays were not performed for strain BG505. Responders are shown in blue and non-responders in purple. (B) On the left, distribution of participants with no neutralizing capacity against any tested strain and with neutralizing capacity against one, two, three, four, five or six strains. On the right, similar plots showing the distribution of participants with detectable gp120-specific IgG or SOSIP-specific IgG. IgG levels were tested against seven strains. (C) Spearman correlations between SOSIP-specific IgG and gp120-specific IgG, both expressed as median fluorescence intensity (MFI), with the Spearman rho correlation coefficients and P-values indicated on each graph. (D) Spearman correlations between half-maximal infective dilution (ID50) pseudovirus neutralization titer and SOSIP- or gp120-specific IgG expressed as MFI. Spearman rho correlation coefficients and P-values are indicated on each graph. (TIF) [file ppat.1012739.s006.tif]

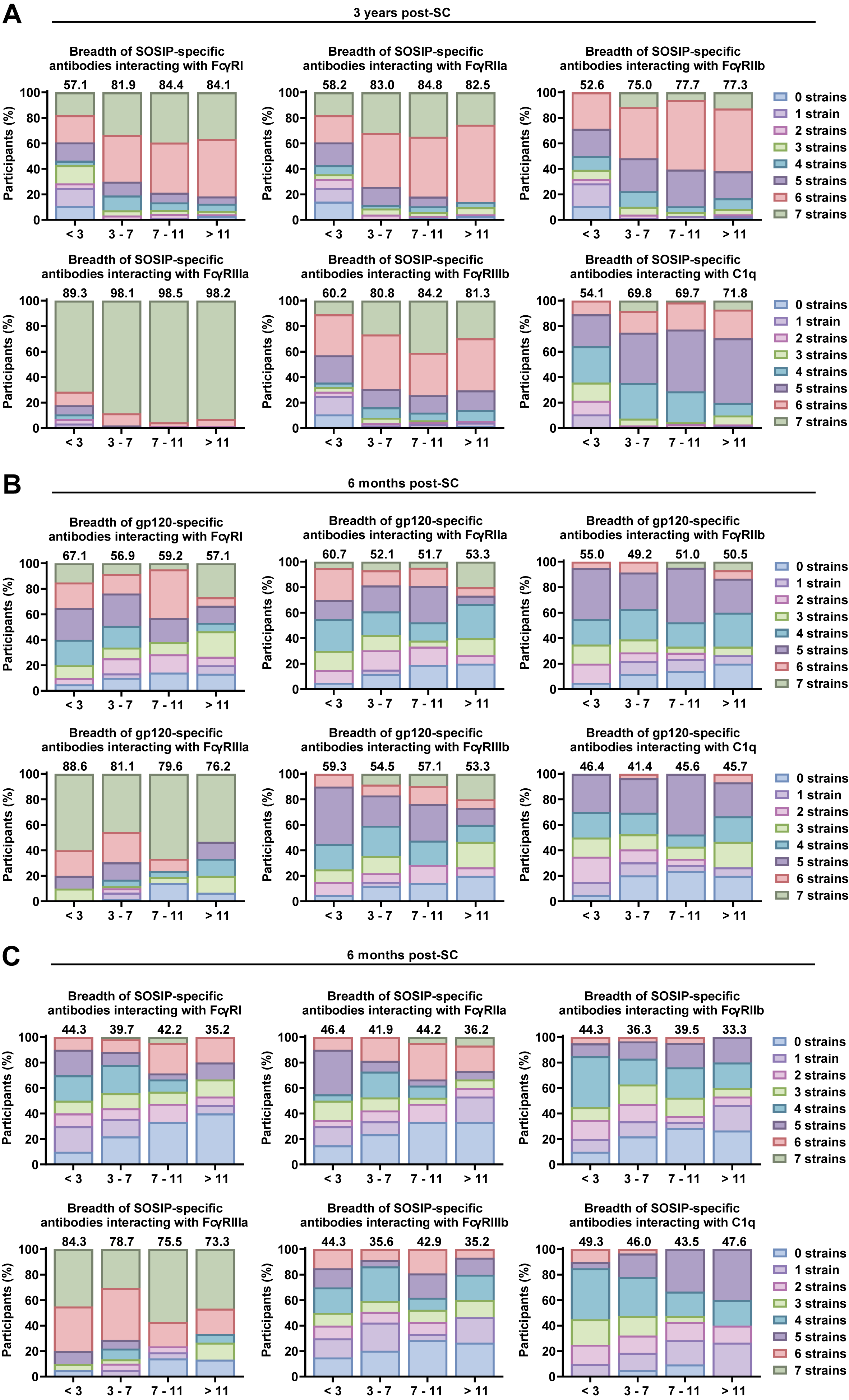

Supplement: S6 Fig — (A) Breadth of SOSIP-specific antibody interaction with FcγRI, FcγRIIa, FcγRIIb, FcγRIIIa, FcγRIIIb and C1q at 3 years post-seroconversion (SC) is compared between four categories based on time between SC and acquired immunodeficiency syndrome (AIDS) diagnosis: less than 3 years (n = 28), between 3 and 7 years (n = 147), between 7 and 11 years (n = 66) and more than 11 years (n = 71). (B) Breadth of gp120-specific antibody interaction with FcγRI, FcγRIIa, FcγRIIb, FcγRIIIa, FcγRIIIb and C1q at 6 months post-SC is compared between four categories based on time between SC and AIDS: less than 3 years (n = 20), between 3 and 7 years (n = 59), between 7 and 11 years (n = 21) and more than 11 years (n = 15). (C) Breadth of SOSIP-specific antibody interaction with FcγRI, FcγRIIa, FcγRIIb, FcγRIIIa, FcγRIIIb and C1q at 6 months post-SC is compared between the same four categories based on time between SC and AIDS. Bar charts show the percentage of individuals within each group with antibody interaction with the plotted FcγR or C1q above the responder cut-off for the color-coded amount of strains. Above each bar the breadth index for that group is shown as a quantitative measure of breadth. This is a value between 0 and 100 calculated with a similar calculation as for polyfunctionality in Fig 2, by weighted addition of the percentage of individuals in each category based on the number of features, derived from Larsen et al.[54]. (TIF) [file ppat.1012739.s007.tif]

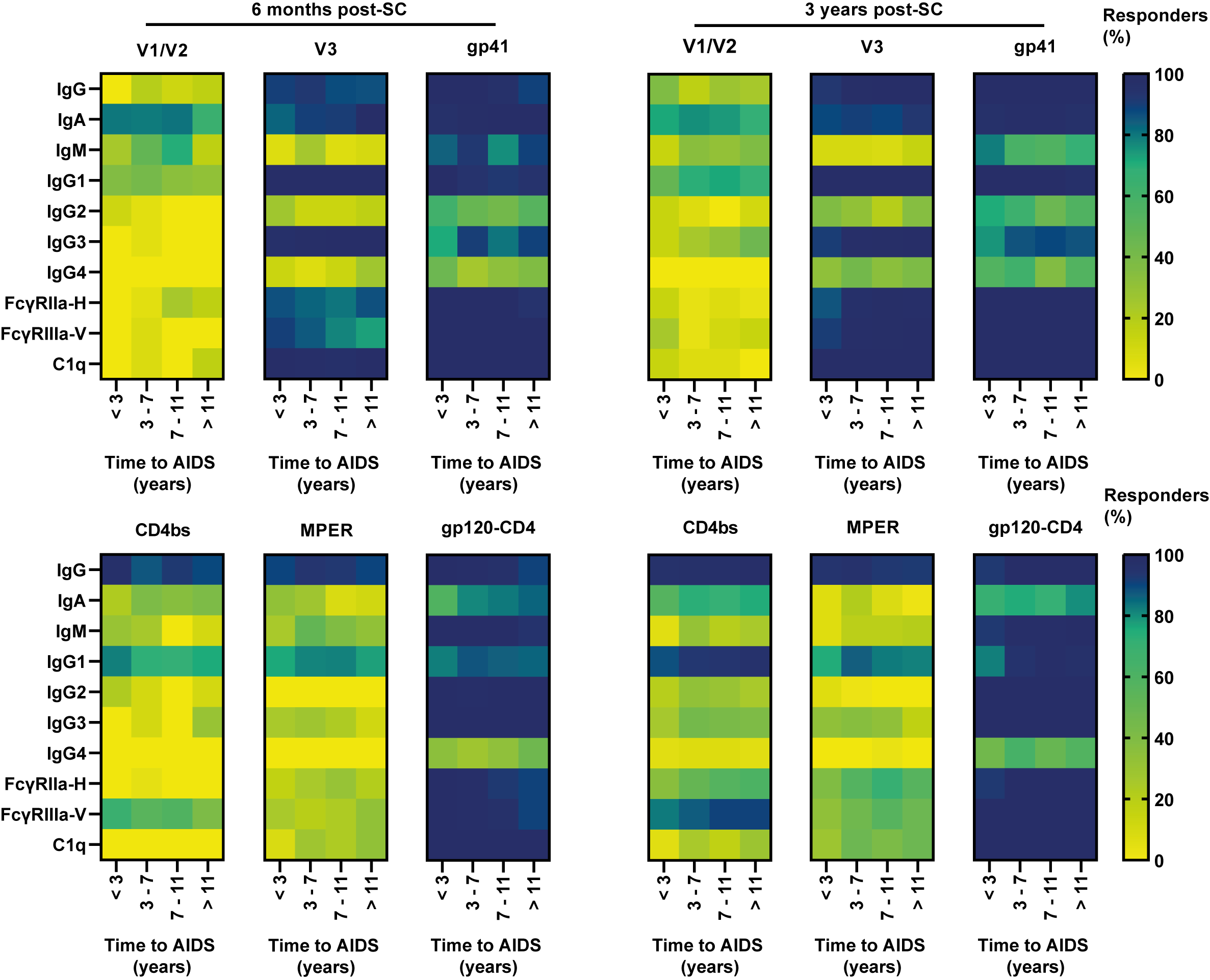

Supplement: S7 Fig — Responder rates in percentage are indicated by the heatmap colors. On the left, in serum of 166 participants at 6 months post-seroconversion (SC), compared between four groups based on time between SC and acquired immunodeficiency syndrome (AIDS) diagnosis (less than 3 years (n = 20), between 3 and 7 years (n = 59), between 7 and 11 years (n = 21) and more than 11 years (n = 15)). On the right, in serum of 382 participants at 3 years post-SC, split based on disease progression in four categories (less than 3 years (n = 28), between 3 and 7 years (n = 147), between 7 and 11 years (n = 66) and more than 11 years (n = 71)). (A) For 15 different 92BR020 SOSIP- and gp120-specific antibody features and (B) for 10 different antibody features specific for six constructs presenting HIV-1 Env epitope clusters. Abbreviations: V1/V1: variable regions 1 and 2; V3: variable region 3; CD4bd: CD4 binding site; MPER: membrane-proximal external region; gp120-CD4: a covalently linked fusion of gp120 and CD4. (TIF) [file ppat.1012739.s008.tif]

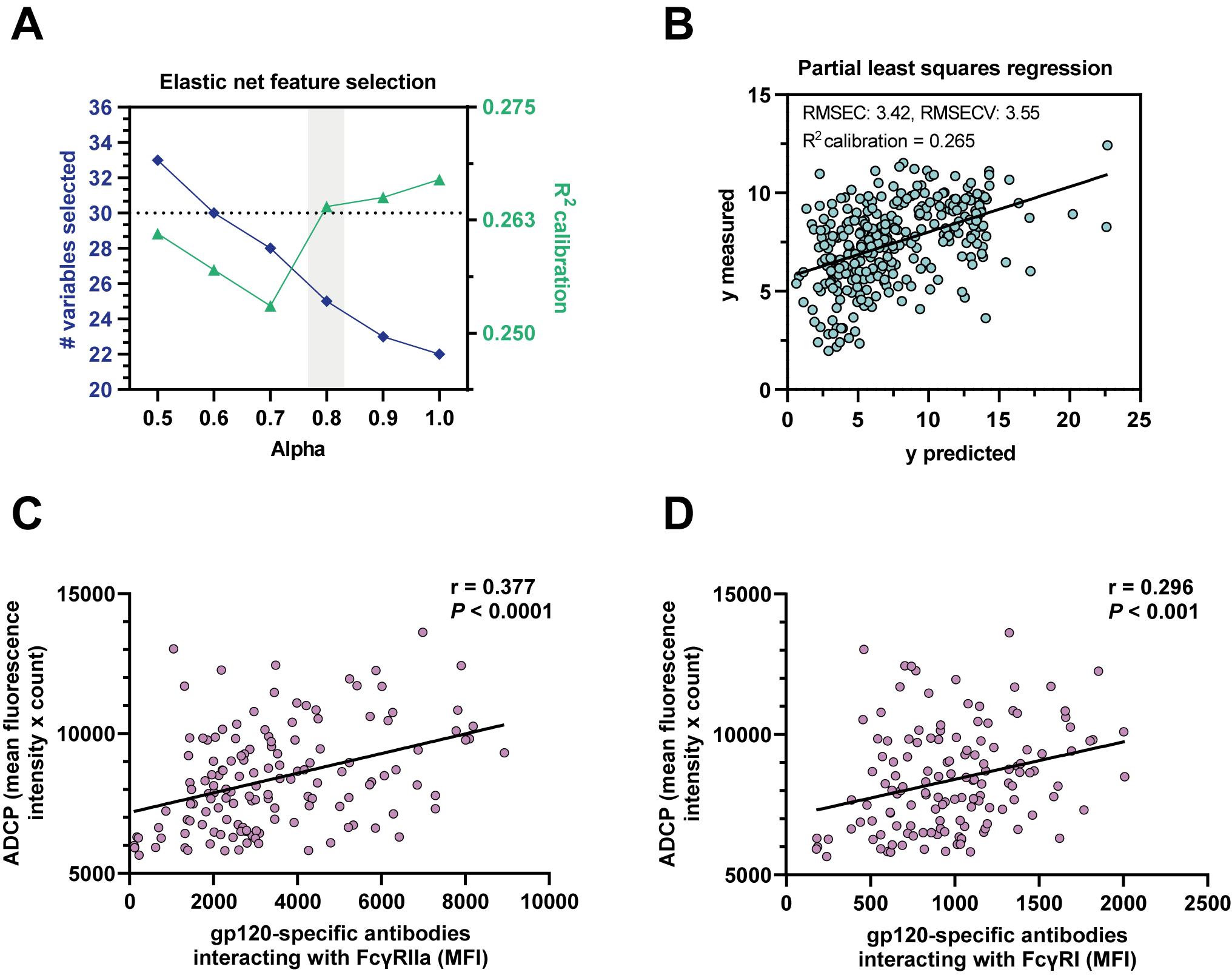

Supplement: S8 Fig — Additional figures for the partial least squares regression analysis for the association between antibody Fc features and disease progression and correlation between ADCP and prominent features (A) Outcomes of six rounds of elastic net feature selection with different values for alpha and 17-fold cross-validation as performed for the partial least squares regression analysis shown in Fig 5. The number of selected variables is plotted on the left y axis and the R2 of calibration of the partial least squares regression analysis is shown on the right y axis. Alpha 0.8 was chosen, as indicated by the grey area. (B) Additional model information for the partial least squares regression analysis shown in Fig 5. Measured values of y are time between seroconversion (SC) and acquired immunodeficiency syndrome (AIDS) diagnosis in years as recorded in the cohort database while y predicted are the predictions of the model. (C) Spearman correlation analysis between antibody-dependent cellular phagocytosis (ADCP) of 92BR020 gp120-conjugated beads by THP-1 cells at 3 years post-SC and 92BR020 gp120-specific antibody interaction with FcγRIIa and (D) FcγRI. The ADCP assays were done on a subset of 140 participants with a similar distribution of time between SC and AIDS as the full cohort. r = rho Spearman correlation coefficient, Abbreviations: RMSEC: Root mean square error of calibration; RMSECV: Root mean square error of cross-validation, MFI: median fluorescence intensity. (TIF) [file ppat.1012739.s009.tif]

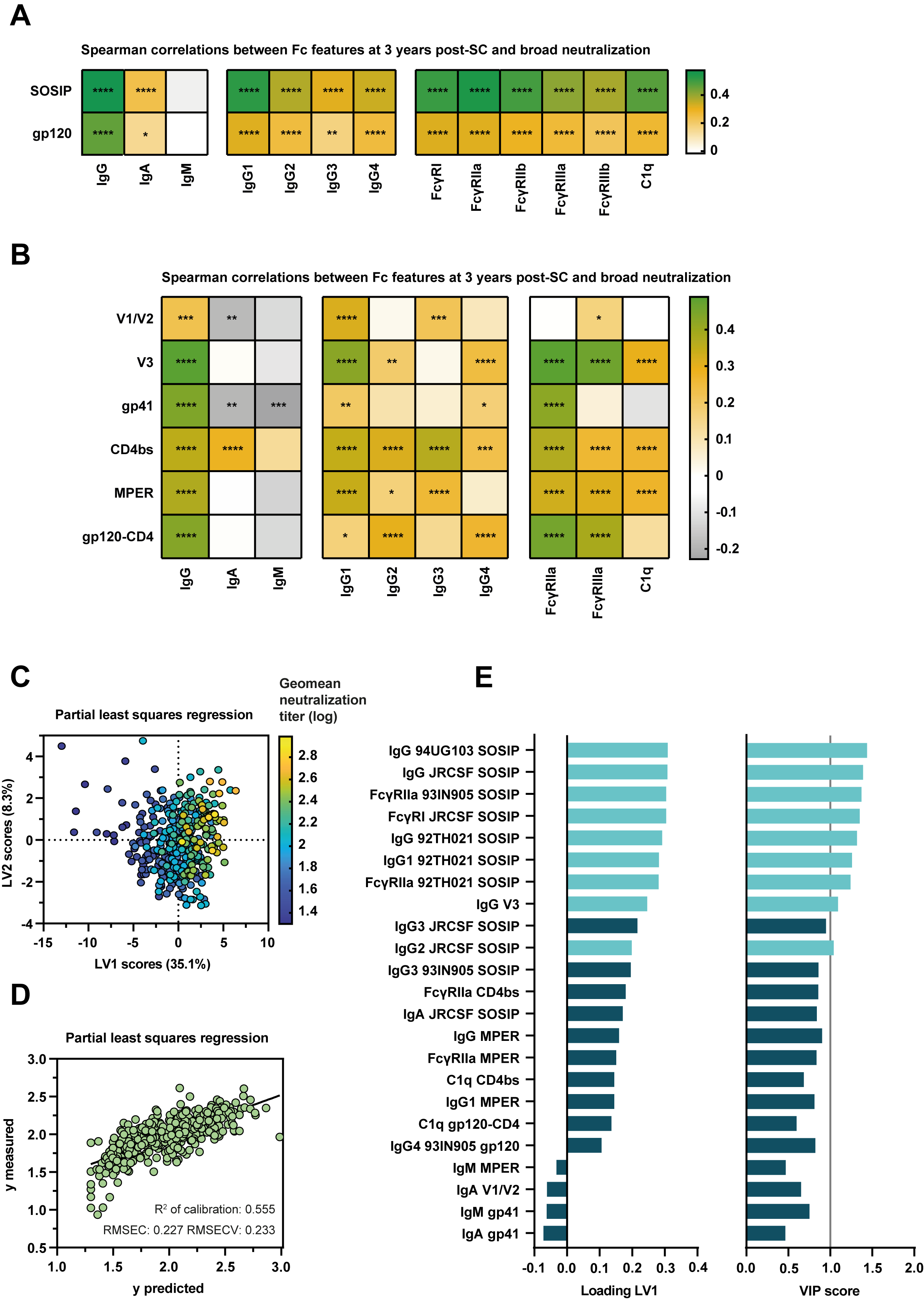

Supplement: S9 Fig — (A) Spearman correlation analysis between the antibody features analyzed in this study at 3 years post-seroconversion (SC) and geometric mean (geomean) half-maximal infective dilution (ID50) pseudovirus neutralization titers at 3 years post-SC (n = 382). Antibody features shown in this figure are for responses specific for gp120 and SOSIP proteins of subtype B strain 92BR020. The color indicates the rho Spearman correlation coefficient and significant results are indicated by asterisks. The results were corrected for multiple comparisons using the Bonferroni-Holm method. (B) Spearman correlation analysis between the antibody features analyzed in this study at 3 years post-SC and geomean ID50 pseudovirus neutralization titers at 3 years post-SC (n = 382). Antibody features shown in this figure are for responses specific for gp120 and SOSIP proteins of subtype B strain JRCSF, V1/V2, V3 and gp41 epitope constructs were also based on the JRCSF sequence. Constructs comprising the CD4 binding site (CD4bs), the membrane-proximal external region (MPER) and a covalently linked fusion of gp120 and CD4 (gp120-CD4) were previously described (see methods). The color indicates the rho Spearman correlation coefficient and significant results are indicated by asterisks. The results were corrected for multiple comparisons using the Bonferroni-Holm method. (C) Partial least squares regression analysis with geomean ID50 pseudovirus neutralization titer as independent variable. Twenty-three Fc features were included in the model as selected by elastic net feature selection with alpha 0.5 and 20-fold cross-validation. The model comprised two latent variables (LVs). (D) Additional model information. (E) Loading and variable importance in projection (VIP) scores of all 23 included features, with variables with a VIP score higher than 1 shown in light blue (also indicated by the grey vertical line) and the remaining variables shown in dark blue. Abbreviations: V1/V1: variable regions 1 and [file ppat.1012739.s010.tif]

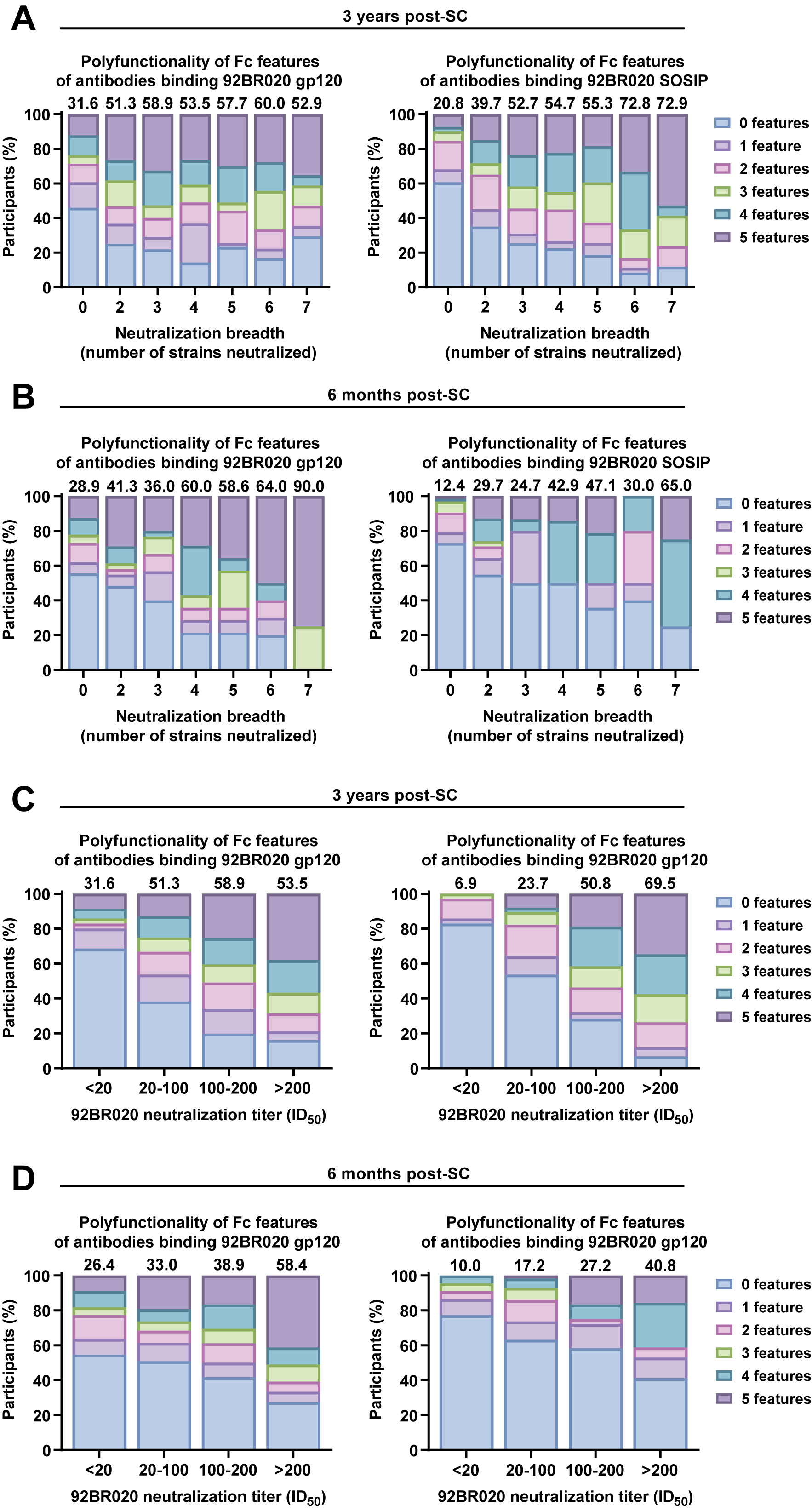

Supplement: S10 Fig — (A) Polyfunctionality was assessed by calculation of a polyfunctionality score at 3 years post-seroconversion (SC) (n = 382). We only assessed activating FcγR (FcγRI, FcγRIIa, FcγRIIIa, FcγRIIIb) and C1q interaction, for the gp120 and SOSIP protein of subtype B strain 92BR020. For each Fc feature we determined for each participant if there was a response that was higher than the median response of all responders in the cohort. The resulting scores (amount of features above the responder median, between 0 and 5) were plotted for seven groups based on neutralization breadth (categorized by the number of strains neutralized). (B) The same analysis was done for antibody Fc polyfunctionality at 6 months post-SC (n = 166). (C) A similar analysis for antibody Fc polyfunctionality at 3 years post-SC (n = 382) but with categories based on 92BR020 half-maximal infective dilution (ID50) pseudovirus neutralization titer. For each Fc feature we determined for each participant if there was a response that was higher than the median response of all responders in the cohort. The resulting scores (amount of features above the responder median, between 0 and 5) were plotted for four groups based on neutralization titer. (D) The same analysis was done for antibody Fc polyfunctionality at 6 months post-SC (n = 166). In addition, a polyfunctionality index was calculated for each group as a quantitative measure of polyfunctionality. This is a value between 0 and 100 calculated by weighted addition of the percentage of individuals in each category based on the number of features, derived from Larsen et al.[54], see methods). This index is shown above each bar. (TIF) [file ppat.1012739.s011.tif]
