# Supplementary material for: Polyfunctionality and breadth of HIV-1 antibodies are associated with delayed disease progression
Source: PLoS Pathog. 2024 Dec 11;20(12):e1012739. doi: 10.1371/journal.ppat.1012739 (PMC11634010; doi:10.1371/journal.ppat.1012739)
Supplement: Attachment — Submitted filename: Response to reviewers.docx [file ppat.1012739.s012.docx]

**Response to reviewers for the manuscript “Polyfunctionality and breadth of HIV-1 antibodies are associated with delayed disease progression”.**

We appreciate the time and effort the reviewers spent to review our manuscript. We are thankful for the constructive feedback, which will improve the quality of the manuscript. We tried to address each of the reviewers comments. The reviewers text is shown in black, our replies are in *italic blue*.

All references to lines in the questions from reviewers are for the numbering in the submitted manuscript.

All references to lines in the answers are for the numbering in the PDF of the revised manuscript including track changes.

**Reviewer #1**

**Summary**

Reviewer #1: In this study, Grobben et al. conducted a considerable analysis to investigate the role of different antibody features on HIV disease progression using samples from a large cohort of untreated people living with HIV. Numerous univariate associations were found between various antibody features, including breadth and polyfunctionality of responses, and delayed disease progression or neutralization breadth/potency. Multivariate analysis indicated FcgRIIA may play an important role. The study is dense with numerous similar analyses which sometimes made it difficult to follow (for instance, Figure 2, S6 and S8 have the same/very similar figure legends) but, overall, the study is an important addition to understanding the many factors that likely contribute to delayed HIV progression and provides further support that polyfunctional antibodies will be important in future HIV vaccine and cure strategies.

**Aspects requiring clarification:**

- The results section (line 120) suggests at least some of this cohort remained ART-naïve up to 11+ years but the method section (study population) is unclear if this cohort remained ART-naïve after the study period ended, stating that participants were ART-naïve until 3 years post-seroconversion (line 526). It seems apparent that these participants remained ART-naïve until AIDS diagnosis or for 11+ years but a clarifying statement would alleviate the concern that ART use at some point during the years post seroconversion.
*All analyses based on time to AIDS were only performed on the time period in which the participants were participating in the cohort in absence of ART. If treatment was started before AIDS diagnosis or treatment was started before 11 years after seroconversion, then participants were not included in these analyses. If participants had no known time to AIDS but participated for at least 11 years with no treatment and without AIDS diagnosis they were included in the analysis, this was done for 44 participants. We have added clarification to the methods section (lines 567-569 and lines 578-582).*

*“AIDS diagnosis in absence of treatment was known for 268 individuals and 44 individuals were not diagnosed with AIDS during their participation in the cohort, but participated in absence of treatment for a least 11 years.”*

*“Some participants stopped participating in the cohort or started antiretroviral therapy before AIDS diagnosis. Individuals who participated 11 years or more without antiretroviral therapy and with no AIDS diagnosis were included in the analyses related to time to AIDS.”*

- It is interesting that the authors decided to undertake the analyses on the full set of participants, including those with genetic factors known to influence disease progression, such as HLA-B57/HLA-B27 and deltaCCR5 mutations. Why were these not removed to avoid these known potential confounders and, perhaps, strengthen the associations in the remaining subset of participants? What was the distribution of these participants in time to AIDS? And did they exhibit any different antibody features to those without known genetic factors?
*DeltaCCR5 and HLA-B57 information was available from 358/383 participants. 73 of those 358 had a CCR5 deletion and 28 of those 358 had HLA-B57. For 277/383 participants HLA-B27 information was available and 23 of those 277 participants had HLA-B27. The distribution of time to AIDS was as follows: deltaCCR5 - median 9,4 (range 2,7-22,6); HLA-B57- median 9,9 (range 3,3-13,4); HLA-B27 - median 10,7 (range 3,3-14,3).We have repeated some analyses after excluding individuals with the mentioned genetic factors known to influence disease progression. In supplementary figure 3, we show that this either has minimal effect on the associations or, mainly for individuals with deltaCCR5 mutations, weakens the associations. This can also be related to the concurrent reduction in sample size. The number of DeltaCCR5, HLA-B57 or HLA-B27 was too low to conduct analysis on these separate groups for differences in antibody features.*

- The majority of binding assays were done using 92BR020 gp120 and SOSIP, but the V1V2, V3 and gp41 proteins used JRCSF. It is clear from the breadth analyses that there are fewer neutralizers against JRCSF than 92BR020, and correlations between neutralization and JRCSF SOSIP binding, as well as between JRCSF SOSIP and gp120 binding, is lower than 92BR020 neutralization and binding, respectively. How similar are the 92BR020 and JRCSF V1V2, V3 and gp41 sequences? It seems possible that lower recognition of JRCSF V1V2 specifically could have contributed to the lack of correlation with progression to AIDS. *The V1V2 sequence between 92BR020 and JRCSF is 65% identical, the V3 sequence is 84% identical and the gp41 sequence is 87% identical. It is known that 92BR020 is a more neutralization sensitive strain than JRCSF. We agree that it is possible that by using a different strain for these epitope constructs we would get different results and we therefore have added a statement about this in the discussion (lines 489-490).*

*“We also observed a difference in antibody binding between strains, therefore the choice of strain for the epitope constructs also likely affects the results.”*

- Similarly, the gp120-CD4 construct, assuming it is FLSC as suggested in figure S12, consists of BaL gp120 which is lab adapted and typically has very high binding (evident in S10). Could this have contributed to decreasing the correlation with disease progression?
*This construct is indeed FLSC, and this is derived from a different strain which likely influences the binding capacity. Therefore, it is possible that these strain differences may affect correlations with disease progression. Similar to the comment above, we have now discussed this in lines 489-490.*

- It is not clear if the comprehensive profiling of antibody features (lines 315-325) is conducted using antibody feature data at 6 months post sero-conversion and 3 years post-seroconversion, or just 3 year post sero-conversion. If both, it does not appear there was any factoring for the dependency/shared effect of that subset of samples? Does this analysis change if using antibody feature data at 6 months or 3 years post-seroconversion separately?
*We have clarified in line 335 that we performed this analysis for 3 years post-seroconversion. We did not perform this analysis for 6 months post-seroconversion, because there were less associations with time to AIDS at this timepoint.*

**Minor issues:**

- Line 191: HLA-B57 (sp.)
*This has been corrected (now line 199).*
- There were no obvious references for the MPER and gp120-CD4 constructs. Line 303 suggests they are in the methods but do not appear to be there.
*These constructs were gifted, therefore this is not mentioned in the protein production section of the methods, but in the section where we describe the coupling of the antigens to the microspheres. Details and references are given in lines 650-656.*

*“In addition to the constructs for which production and purification was described above, we received two HIV-1 antigens through collaborators. The full-length single-chain, a chimeric construct composed of BaL gp120 fused with a flexible linker to the N terminus of the outer domains of CD4, was kindly provided by George K. Lewis from the University of Maryland School of Medicine[93]. A scaffold based on the epitope of MPER antibody 2E5 generated by rational computational engineering was kindly provided by William R. Schief from the Scripps Research Institute[94].”*

- The gp120-CD4 construct is labelled FLSC in Figure S12. Change for consistency.
*This has been changed, and due to other changes this figure has been moved to the online repository).*
- Figures S7C and 7D are switched in the text (lines 233-235). In Figure S7, C refers to correlation between neutralization and SOSIP binding while D refers to SOSIP and gp120 binding.
*Panel C and D have been switched in Figure S7 to correct this error (now Figure S5).*
- Lines 282-283: the increased gp120-CD4 binding by antibodies that bound C1q at < 7 years, otherwise binding seems comparable for > 7 years?
*This text was describing the differences between the plots for C1q, FcγRIIa and FcγRIIIa in supplementary Figure 12 (which has now been moved to the online repository) so it is not related to time to AIDS. Since this is complex and easily confusing, and our main focus was the data in relation to disease progression, we have removed this text entirely (lines 296-301).*
- Line 682: Bonferroni-Holm test (sp.)
*This has been corrected (now on line 742-743).*
- Supplementary table 1 part 1 and 2 (samples at 3 years and 6 months post-seroconversion available): “time since SC” states years but is actually months. Same in Part 2.
*This has been corrected.*

**Reviewer #2**

**Summary**

Reviewer #2: This study uses a data dense methodology to unpick Fc binding and isotype antibody signals associated with the development of HIV neutralization breadth and slowed disease progression to AIDS. While i believe this manuscript to offer unique insights, I find several of the findings overstated and based on weak correlations. I also suggest that some functional data would greatly strengthen the publication and given the expertise of the authors included should not be onerous to perform, especially given the emergence of FcyR2A as a major signature. Several minor suggestions and discussion points are included.

**Part II – Major Issues: Key Experiments Required for Acceptance**

Reviewer #2: 1. I recommend that functional data be included to at least support the breadth associations for FcyRIa and FcyRIIa(ADCP) with slowed disease progression. While interesting that FcyRIIa is a major signature, it is not the only determinate of ADCP and as such to make the claim that ADCP is associated with protection this should be tested, if only in a subset.
*We agree that this is an interesting addition to the study and we have performed ADCP assays on a subset of 140 participants. The results have been added in Figure 5D and the correlations with FcγRIIa and FcγRI are shown in Supplementary figure 8C and D.*

**Part III – Minor Issues: Editorial and Data Presentation Modifications**

Reviewer #2: 1. Abstract: The authors state that “the capacity to interact with all Fc γ receptors (FcγRs) and C1q, and in particular with FcγRIIa, correlated positively with delayed disease progression”. All Fc receptors would also imply FcγRIIb which is predominately inhibitory?
*Indeed, we also found positive associations between FcγRIIb interaction and delayed disease progression. This is now further discussed in the discussion in lines 437-441, and the implications of this outcome are now mentioned in lines 542-543.*

*“Interestingly, the inhibitory FcγRIIb is also part of the profile of antibody features associated with reduced disease progression, indicating that a balanced response may also be important. Moreover, this is also interesting since it was shown that FcγRIIb responses may be associated with improved affinity maturation in the context of Influenza virus [62, 63].”*

*“In such an approach, the affinity for inhibitory FcγRIIb should likely also be considered to achieve a balanced response.”*

2. Introduction: Rephrase the sentence in line 66: “The effectivity of antibody effector functions is determined by a range of factors.” The term effectivity is a strange one which is used repeatedly.
*We thank the reviewer for pointing this out, we have changed all occurrences of the word ‘effectivity’ to ‘effectiveness’.*

3. In line 85 you can also add SARS-CoV-2 – interesting new preprint from Florian Krammer 10.1101/2024.02.28.582613
*This has been incorporated in the text (now on line 89).*

4. The paragraph from line 100 feels repetitive, I think the rationale is clear without multiple repeating lines.
*Lines 107-110 were removed to make the paragraph less repetitive.*

5. It should be made clear that the authors are not looking at Fc effector functions – They are measuring Fc receptor and complement binding as proxies for the actual assays. Albeit they typically good proxies, much of what is known about Fc receptor binding is not always a perfect correlation with the functional output. Further, neutralization is an assay measure of function and the Fc array a measure of binding.
*Indeed this was not very clear, therefore we have adjusted the text in lines 112-115 to reflect this more accurately.*

*“Using 20 different HIV-1 antigens, we assessed the association between 15 different antibody Fc properties and disease progression as well as the coordination between these antibody Fc properties and with neutralization capacity in polyclonal serum.”*

6. It is not clear what is meant by polyfunctionality in this study given no functions were tested, perhaps another term is more correct?
*In this study we define polyfunctionality as high interaction with multiple receptors/proteins that are known to be related to functionality (interaction with FcγRI, FcγRIIa, FcγRIIIa, FcγRIIIb and C1q). However we agree that, with the exception of ADCP assays (as shown above), no other cell-based functional assays were conducted in this study. Therefore, we have more carefully rephrased and clarified this description on lines 208-213.*

*“Antibody Fc polyfunctionality has been associated with HIV-1 control[48]. Therefore, we aimed to predict Fc polyfunctionality for each individual in our cohort by calculating a Fc polyfunctionality score. This score was based on the capacity of their antibodies to interact with multiple activating FcγRs (FcγRI, FcγRIIa, FcγRIIIa, FcγRIIIb) and C1q, given that these interaction levels were previously demonstrated to highly correlate with a range of different Fc functions in cell-based assays[51-53].”*

7. I am not clear as to why such detailed characterisation is focused on 92BR020 SOSIP. Appreciating that this in itself is a lot of work, if it’s because it has not been previously characterised then this should be clarified in the text.
*This is indeed because it was produced specifically for this study and it was not previously characterized. We have added this in the method section to lines 609-611.*

*“The newly produced SOSIP proteins were characterized using blue native PAGE, negative-stain electron microscopy, and by assessing binding of a panel of well-characterized monoclonal antibodies.”*

8. Do the non-responders in the SOSIP groups for Figure 1 correlate with neutralization against the matched virus?
*Figure 1 does not differentiate between responders and non-responders, but in Supplementary Figure 5 we did compare neutralization and IgG responses for each virus in panel D. We now clarified that this concerned the matched virus in the results in line 247.*

*“Moreover, IgG binding results at 3 years post-SC correlated significantly with the previously obtained neutralization potency results against the matched virus at the same time point (Sup. Fig. 5D).”*

9. Sup Fig 2: it is not clear how the authors decided on which features to show in the main text and which to show in the supp?
*The features shown in the main figure are those we were most interested in at the start of the study, based on information described in previous literature such as which functions have been associated and which roles have been described for each feature. This was the reasoning to show the subtypes of IgG in the main figure as well as total antigen-specific IgG, FcγRIIa (associated with ADCP), FcγRIIIa (associated with ADCC) and C1q (associated with activation of the complement system). This has now been clarified in the legend of Figure 1 (lines 164-165).*

*“The eight features shown here are those we were most interested in based on literature, seven additional features are shown in Sup. Fig. 2A.”*

10a. Sup Fig 2B – on the figure it would be helpful to have a key for “time to AIDS” *Additional keys have been added to supplementary figure 2 and 7.*

10b. also from Fig 1A in the main text seems like everyone has IgG3 levels but this figure doesn’t show that?
*The graphs in Fig 1A are meant to compare the IgG3 levels between the two timepoints. whether these responses are defined as ‘responders’ or not. In Supplementary Figure 2B, the responder rate is shown. This is based on the MFI, the negative control sera and the beads without antigen. There was a very high responder threshold for IgG3 which we unfortunately failed to notice. This was skewed by one healthy donor individual with a very high signal for IgG3 binding 92BR020 gp120 and SOSIP. This is a very clear statistical outlier (equal to Q3 + approximately 350x the IQR). Therefore, we deemed it appropriate to remove this outlier from the calculation, resulting in a responder cut-off for IgG3 which appears similar to the others. Sup Fig 2B has been corrected accordingly and this correction is now also mentioned in the method section (lines 710-711).*

*“One major outlier HIV-1-negative individual had to be excluded from these calculations for IgG3.”*

11. Line 177: double full stop
*This has been corrected.*

12. The correlations in 1B, although significant are extremely weak and do not show a particular meaningful correlation – in the results the authors state that these are strong which should be corrected. As such it is difficult to suggest that these features are associated with disease progression
*We agree that the correlations should not be called strong, this has been corrected (line 185). The words ‘are associated’ have also been replaced by ‘correlate’ in line 176.*

13. While the polyfunctionality score is clear , it is not clear what the polyfunctionality index shows that is more informative than the score.
*The polyfunctionality index is a more quantitative score to assess polyfunctionality, encompassing all possibilities for the amount of features in one number rather than a score for each possibility which needs to be shown in a stacked graph to be interpreted.. A sentence was added to line 225, 264, 1165, 1210 and 1306 to clarify this.*

*“In addition, a polyfunctionality index was calculated for each group as a quantitative measure of polyfunctionality.”*

14. Authors state that 25% of these participants were previously determined to have a bNAb response (defined as neutralizing >4 out of 6 strains with an ID50>10047) . This should also make reference to the Simek paper that defined this downselected panel.
*The reference has been added to line 243.*

15. In general there are too many supplementary figures and I found this made reading the study overwhelming, I suggest decreasing non-essential data
*Five supplementary figures have been moved from the supplement to an online repository.*

16. It is stated that the neutralization titer correlated better with SOSIP than gp120, as expected but the correlation with many of the timers is weak. Given that several of the worst correlating trimers were also those with aggregation, is this data against these trimers reliable and representative of trimer binding?
*There could be several reasons why the correlations are weaker. One of which is the aggregation which we observed when we characterized the trimers. However, probably a larger factor is the strains that were tested. Since all participants were subtype B infected, it is also not surprising that the correlations with subtype B strains 92BR020 and JRCSF are stronger. In addition, when we tested all SOSIP timers with a panel of monoclonal antibodies, we did not see clear differences between the trimers that aggregated and the primers that were not. To clarify, we added a sentence describing this in the results section in lines 237-238.*

*“However, this did not appear to influence the patterns of recognition by a panel of monoclonal antibodies specific for different HIV-1 epitopes (Sup. Fig. 1C).”*

17. For the constructs representative of different epitopes, the strains from which they were derived should be stated in the main text. The association with slowed disease progression should also be tempered here with the statement that only specific strains were used.
*For each construct, the strain is now mentioned in the main text in lines 274-277. In addition, we re-wrote the results to more specifically mention that we investigated antibody binding to our constructs to indicate that that this likely does not completely encompass all possible antibodies that bind these epitopes (lines 293-35).*

*“Therefore, we also analyzed whether antibody responses could recognize constructs presenting the following epitopes: the V1/V2 region (strain JRCSF), the V3 region (strain JRCSF), the CD4 binding site (derived from strain HXB2), gp41 (strain JRCSF) and the membrane-proximal external region (MPER, based on the 2E5 antibody epitope). In addition, we included a gp120 monomer fused to soluble CD4 (gp120-CD4; strain BaL) to assess antibodies that recognize CD4-induced (CD4-i) epitopes, which were previously associated with antibody-mediated effector functions[58].”*

*“Antibody binding to these MPER and V1/V2 constructs was relatively low for antibodies interacting with FcγRIIa, FcγRIIIa and C1q, while JRCSF gp41-specific antibodies frequently interacted with FcγRIIa, FcγRIIIa and C1q (data included in the online repository). SOSIP- and JRCSF V3-specific antibodies interacting with C1q were slightly increased in individuals with slower disease progression (Fig. 4B). At 3 years post-SC, there was also an increase in gp120-, gp120-CD4-, V3- and gp41 construct-specific antibodies interacting with FcγRIIa, while the largest differences were observed for FcγRIIIa, with increased levels of gp120-, gp120-CD4-, SOSIP- and V3 construct-specific antibodies in individuals with slower disease progression.”*

18. Again the correlations shown in Figure 4a are significant but weak, as such conclusions should be tempered.
*We adjusted the title of this section (line 270). Conclusions are tempered in the discussion (line 496).*

19. Are the differences shown in Figure 4B significant? Again these seem very slight.
*Figure 4B is a comparative analysis. By normalizing across the different antigens, we can visualize differences and where there is most effect related to time to AIDS and compare this between the three antibody features. Differences can appear slight because of the normalization. We performed Kruskall-Wallis tests on the data (pre-normalization), and have now added the results of that comparison to figure 4B to clarify statistical significance.*

20. The authors state “Breadth was not identified as important, whereas polyfunctionality did assume a clear role in the model.” Can this be clarified?
*This refers to the fact that all our breadth variables were not selected during feature selection and several polyfunctionality variables were selected as feature. Of which, one polyfunctionality variable had a VIP score higher than 1. To clarify this, we have edited a sentence in lines 340-341.*

*“Breadth was not selected, whereas polyfunctionality did assume a clear role in the model.”*

21. Discussion: While interesting that FcyRIIa is a major signature, it is not the only determinate of ADCP and as such to make the claim that ADCP is associated with protection this should be tested, if only in a subset. Further the authors go on to mention that FcyRIIa binding could be improved in bNAbs but perhaps more mention should also be made to FcyIIB interaction which would also have to be considered to balance out effects
*As mentioned above, we have performed ADCP assays on a subset of 140 participants. The results have been added in Figure 5D and the correlations with FcγRIIa and FcγRI are shown in Supplementary figure 8C and D.* *Indeed, it is also interesting that FcγRIIb is also part of the profile associated with reduced disease progression in our study. We have now described this in the discussion in lines 437-441. And also added a sentence about this in lines 542-543.*

*“Interestingly, the inhibitory FcγRIIb is also part of the profile of antibody features associated with reduced disease progression, indicating that a balanced response may also be important. Moreover, this is also interesting since it was shown that FcγRIIb responses may be associated with improved affinity maturation in the context of Influenza virus [62, 63].”*

*“In such an approach, the affinity for inhibitory FcγRIIb should likely also be considered to achieve a balanced response.”*

22. Is the association seen with IgG1 just a titer effect? Ie it is just associating so strongly because there is more in the plasma. This may be relevant given that the author later postulates that they may have missed V1/V2 associations etc because of titer.
*All associations in our study are indeed to some extent influenced by titers. However, with sufficient titers, as is the case for most of the features we analyzed, we expect to see associations if they are present. Only for the V1/V2, MPER and the CD4-binding site construct, the titers are unfortunately very low and may have caused us to miss potential associations. This is discussed in lines 484-489. The distribution of that data can now also be viewed in graphs which will be placed in the online repository.*

*“Importantly, there were large differences between the quantity of antibodies recognizing the different epitope constructs. Therefore, the strength and statistical significance of the associations for the different epitopes are likely influenced by the relative abundance of antibodies with certain specificities. Associations for highly abundant antibody specificities may be overemphasized while it is also plausible that we missed potential associations because of the lower titers for V1/V2, the CD4bs and the MPER.”*

23. The authors state that their study is unique because they are unpicking the mechanisms of slowed progression but they go on to say that nothing can show causality – perhaps in the introduction the authors show strengthen the novelty of their study.
*We understand this comment, and we have re-written the last two paragraphs of the introduction (line 103, lines 107-115). The text is now more clear about how we mostly elucidated a more clear antibody profile, and highlights other features that make our study unique.*

24. Why were the associations for IgA were limited in your analysis?
*We measured IgA levels to all antigens and included this in quite a few of our analyses, See Supplementary Figure 2, 7, 9, main Figure 5 and 6 and the online repository. However, we did not find many associations for this antibody type. Perhaps the word limited was confusing, this has been changed to ‘not frequent’ in line 461.*

25. “We found that antibodies with functional Fc features, especially when interacting with C1q more often recognize CD4-i epitopes and the V3 region” – can the authors postulate as to why?
*We can postulate, for example that the orientation of the antibodies plays a role. Since these regions are occluded in the native envelope state and are only accessible after CD4 engagement, the orientation of the bound antibodies could favor interaction with C1q and FcγRs. This could be further enhanced by the structural and spatial preference of the hexameric C1q molecule to engage with multiple antibody Fc tails in close proximity. However, we think this is highly speculative and cannot prove these hypotheses, therefore we did not include such statements in the manuscript.*

26. More mention should also be made of the fact that the strains of v3 , v2 etc are limited and may contribute to skewing of the patterns seen.
*This has been added to the discussion in lines 489-490.*

*“We also observed a difference in antibody binding between strains, therefore the choice of strain for the epitope constructs also likely affects the results.”*

27. The observation that Fc is associated with neutralization breadth but also slowed disease progression is interesting but in the discussion, the differences in signatures should be more clearly contrasred. The association of any SOSIP binding titers is expected but it is interesting that FcyR2a v3 is a signal for breadth when v3 binding mAbs are often a hinderance for breadth.
*We have contrasted our findings for disease progression and neutralization breadth in the discussion already in lines 493-509. Indeed it is interesting that the V3-specific FcγIIa interaction is part of the signature associated with neutralization breadth. We added this to lines 501-503.*

*“That antibodies can both mediate effector functions and have neutralizing activity is now established, yet there is currently not much emphasis on measuring the induction of antibodies with both functionalities. In our analysis, we identified that early antibody responses including IgG1, antibodies with the capacity to engage FcγRIIa, FcγRIIIa and C1q are predictive for the development of antibodies with broad neutralization at 3 years post-SC. This complements a previous study which found that polyfunctional antibodies, FcγR binding antibodies, antibody-dependent cellular trogocytosis and antibody dependent complement deposition early in HIV-1 infection were associated with the development of neutralization breadth[59]. It was suggested that this indicates possible shared underlying factors for both Fc functionality and neutralization breadth. We also observed an unexpected association between neutralization breadth and antibodies targeting V3 and gp41. In our study, early heterologous SOSIP-specific responses were also predictive for broad and potent neutralization activity, but these responses did not associate with disease progression. It is plausible that the strong correlations we observed between antibody Fc features and neutralizing antibodies at 3 years post-SC can be explained by the IgG1 titers in our cohort. However, the previously established lack of an association between (broad) neutralization and delayed disease progression, indicates that the Fc functional properties of this antibody response are likely crucial for the observed association with delayed disease progression.”*

28. Were the bNAbers matched in viral load for non-bNAbers, as a major driver of breadth this is important to mention.
*We did not match any of the participants in any way, and worked with the full cohort where possible. Analyses including viral load and the development of bnAbs were previously done in Euler et al. 2019 doi:* *https://doi.org/10.3390/v11040384. A statement was added to confirm that we did not do any matching in lines 578-579.*

*“These are described in Table 1 and Supplementary Table 1, no matching was performed.”*

29. In light of the data for the association with breadth the authors may consider changing the title to encompass this finding
*Since the major focus of our study was to explore antibody Fc features that associate with disease progression, we would prefer to keep the current title. However, we agree with the reviewer that the association with breadth is very interesting, therefore we have highlighted this more clearly in the abstract (lines 11 and 16-17).*

30. The final conclusion “In conclusion, our study identifies correlates for prolonged control of HIV-1 disease progression” should be tempered given weak associations and rather focused on the second half of that statement “provides a specific biophysical profile of antibody features attractive for preventative and therapeutic strategies against HIV-1”. *The final conclusion has been adapted (line 551-553).*

**Reviewer #3**

Advances state of the are in particular b/c it includes SOSIP. Really fascinating correlative relationships in Figure 1B. Suggest to consider showing one raw scatterplot.

*Since the supplementary materials are already extensive, we chose to include this in an online repository instead. We have now included scatterplot graphs of all data underlying figure 1B in the repository.*

Line 91 – what are the “several studies”? they should be cited.
*The studies are further explained and cited in the lines following that sentence (line 98-101). Lines 95-96 have been adapted to make this more clear.*

References could be more balanced and extend further back in time.
*We have added a few more references to the manuscript, including some of the earlier work on the association between antibody effector functions and HIV-1 disease progression. We have added references 11, 12, 13, 42, 49, 51, 52, 53, 62 and 63 in response to this and other comments.*

There seem to be a lot of methods details in the results.
*We understand this comment. This is because we found it necessary to be clear about our approach to allow the readers to follow the manuscript, since there is a lot of data. In addition, we produced quite some novel protein antigens as part of the study and these proteins and their characterization is also a result of the study.*

Fc Array method should be cited.
*A reference has been added to line 145.*

Suggest to add fold change information (and baseline or negative population values) in Figure 1A. *The reason we refrained from adding fold-changes to figure 1A is that the data is not completely linear. Therefore, we do not feel that it is representative of the data if we add fold-changes to this figure since the fold-changes would be larger when the data is in the linear range of the assay. Therefore, we found that comparisons using nonparametric tests would be more suitable.
The negative population results have been used to calculate responder cut-offs, this gives an indication of the percentages of responders in supplementary figure 2B. This contains the information that could be acquired from adding negative population values to Figure 1A and that is why we decided not to make Figure 1A more complicated by adding this. The underlying data will be made available in the online repository.*

Suggest to specify timepoint in figure 1(B) legend. You say another timepoint is in supplemental, but not which TP is shown in legend, though it is described in the text.
*This information has been added to the legend of figure 1B in line 166.*

Can the authors be sure that some responses are not saturated under the experimental conditions used? Data for some features (IgG1 SOSIP) suggests that it may be.
*The data is not all in the linear range of the assay, but it is also not saturated. We found that displaying the data on a logarithmic scale was most appropriate and gives the best view of the data. The data in the upper range of the assay however, appears to near saturation on the logarithmic scale, but this is not the case when displayed on a linear scale. The serum dilutions used in the assay have been carefully optimized to prevent saturation and also obtain as much information as possible in the lower range.*

How were thresholds defined for determining responder/non-responder?
*The method used to determine responders and non-responders is explained in the methods section in lines 706-712.*

*“We determined a cut-off for a detectable antigen-specific antibody response for each antigen individually using sera of 10 HIV-1-negative individuals and the no protein control bead. This was as follows: a response above 100 MFI, more than 3x the MFI of the no protein control bead in the same well and above the 95th percentile of the measurements of the 10 HIV-1-negative individuals for the same antigen. One major outlier HIV-1-negative individual had to be excluded from these calculations for IgG3. The sera of the HIV-1-negative individuals were always included on the same plate as the cohort samples. Responder cut-offs were the same for the samples at 6 months and 3 years post-SC.”*

Other groups frequently use geometric means to score Ab breadth. Can the authors investigate the effect of that scoring choice on their observations?
*Indeed geometric mean is a common method to assess breadth. We did use this method, in figure 6 and supplementary figure 9.*

A figure legend doesn’t seem an appropriate place for mathematical formulas.
*The formula was removed from all figure legends (lines 227-228, 266-267, 1167-1168, 1213-1214 and 1308-1309)*

Line 277-281 – it seems (potentially) misleading to state that MPER and V1V2 responses were virtually undetectable on the basis of normalized data. In general, many groups hesitate to compare responses between antigens using Luminex/MSD/ELISA-style measures because each antigen may present different linear ranges, dose-response profiles, etc. As a result, such comparisons may reflect technical assay differences more than biologically interesting differences. As a general comment, making sure where relative, z-scored, raw, other data inputs are analyzed across the manuscript is clear is an important aspect for the authors to confirm.
*Indeed the sentence that these responses were virtually undetectable was incomplete and not correct. It has been adapted to state that the response was relatively low, and clarified that this was the case for FcγRIIa, FcγRIIIa and C1q speficially (lines 293-296).*

*“Antibody binding to these MPER and V1/V2 constructs was relatively low for antibodies interacting with FcγRIIa, FcγRIIIa and C1q, while JRCSF gp41-specific antibodies frequently interacted with FcγRIIa, FcγRIIIa and C1q (data included in the online repository).”*

PLS analysis needs a negative control (ie. Prediction on permuted time to infection). Methods lack sufficient detail. For example, cross-validation was described, but it was not clear if the results of held out samples were the same as those that were used in training. Because repeated cross-validation is not described, the reader is left to presume that the model is more of a fitting exercise than a prediction, as only 5-8% of the data is held out with 12-20-fold cross-validation. If the authors scramble input data or the predicted/fit outcome variable and observe similar results, claims about the biological relevance of the model features and goodness of fit would need to be tempered/eliminated. If cross-validation was only used to select the identity/number of features, this should be made clear and it would still make sense to conduct permutation testing with that modeling approach.
*We have now run a Probability of Model Insignificance vs. Permuted Samples analysis as a negative control. This analysis indicates that the predictions for our model are significantly different from permuted models, P<0.001 by Wilcoxon test. This has been included in the methods section in lines 755-758.*

Line 405 - When discussing the dominance of the role of IgG1 it would be appropriate to note that, at least based on prior studies, if not absolutely quantified here, the majority of the HIV-specific IgG is IgG1, thus it would be expected to exhibit a dominant role.
*Indeed, we agree that the fact that most IgG is IgG1 should be mentioned as it is certainly expected to be a big factor in why it has a big role. The sentence has been adapted (lines 434-437).*

*“The dominance of IgG1 antibodies, the association of IgG1 with delayed disease progression and the observed associations between IgG1 and antibody features such as FcγRIIa interaction, indicate that IgG1 is likely most responsible for this effect in our cohort.”*

Line 418-419 – suggest to revise. I think the authors may mean to say that other factors may play a mechanistic role in the observed associations (eg. with progression). If they mean something else, it would be helpful to revise the sentence to make that clear.
*Indeed, we wished to point out the possibility that these association could be indirect, if the factors we investigated are linked to other factors that do play a mechanistic role. The sentence has been revised (lines 452-454).*

*“However, these studies are unable to prove causality and therefore it remains possible that these are indirect correlations, linked with other factors that do play a mechanistic role in the observed associations.”*

Line 432 – I’m not sure IgG3 halflife is a relevant explanation of serum IgG3 levels over time in the context of chronic infection (and antigen stimulation).
*This is indeed disputable. Since our main point was to mention subclass switching as a possible cause, we have omitted the sentence discussing IgG3 half-life (lines 465-466).*

Appreciated thoughtful caveats described starting at like 450.
*Thank you for the appreciation.*

Feels almost negligent not to mention failures of recent vaccine efficacy field trials recent vaccine concepts that prioritized effector function, especially since the authors seem to suggest that there should be more emphasis on these activities. Perhaps they mean to suggest that the pendulum has swung too far into interest in neutralizing activity alone?
*We did not intend to focus on potential applications for vaccination, indeed due to the lack of success in vaccine trials that tried this approach. Nevertheless, more information on this topic would be useful for those that are pursuing this strategy. The fact that this has been tried and did not succeed yet is now mentioned in lines 48-49.*

*“However, vaccine efficacy studies focused on antibody-mediated effector functions also have not yet led to clinical success.”*
